# Impact of Circulating Cell-Free DNA (cfDNA) as a Biomarker of the Development and Evolution of Periodontitis

**DOI:** 10.3390/ijms24129981

**Published:** 2023-06-10

**Authors:** Gaia Viglianisi, Simona Santonocito, Alessandro Polizzi, Giuseppe Troiano, Mariacristina Amato, Khrystyna Zhurakivska, Paolo Pesce, Gaetano Isola

**Affiliations:** 1 Department of General Surgery and Surgical-Medical Specialties, School of Dentistry, University of Catania, 95124 Catania, Italy; gaia.viglianisi@gmail.com (G.V.); simonasantonocito.93@gmail.com (S.S.); alexpoli345@gmail.com (A.P.); gaetano.isola@unict.it (G.I.); 2 Department of Clinical and Experimental Medicine, University of Foggia, 71122 Foggia, Italy; khrystyna.zhurakivska@unifg.it; 3 Department of Surgical Sciences and Integrated Diagnostics (DISC), University of Genoa, Ospedale S. Martino, 16148 Genoa, Italy

**Keywords:** periodontitis, circulating cell-free DNA, oral disease, periodontics, tooth loss: oral cancer, trials

## Abstract

In the last few decades, circulating cell-free DNA (cfDNA) has been shown to have an important role in cell apoptosis or necrosis, including in the development and evolution of several tumors and inflammatory diseases in humans. In this regard, periodontitis, a chronic inflammatory disease that can induce the destruction of supporting components of the teeth, could represent a chronic inflammatory stimulus linked to a various range of systemic inflammatory diseases. Recently, a possible correlation between periodontal disease and cfDNA has been shown, representing new important diagnostic–therapeutic perspectives. During the development of periodontitis, cfDNA is released in biological fluids such as blood, saliva, urine and other body fluids and represents an important index of inflammation. Due to the possibility of withdrawing some of these liquids in a non-invasive way, cfDNA could be used as a possible biomarker for periodontal disease. In addition, discovering a proportional relationship between cfDNA levels and the severity of periodontitis, expressed through the disease extent, could open the prospect of using cfDNA as a possible therapeutic target. The aim of this article is to report what researchers have discovered in recent years about circulating cfDNA in the development, evolution and therapy of periodontitis. The analyzed literature review shows that cfDNA has considerable potential as a diagnostic, therapeutic biomarker and therapeutic target in periodontal disease; however, further studies are needed for cfDNA to be used in clinical practice.

## 1. Introduction

Periodontitis is a chronic disease that affects 10% of the world’s population [1]. It is characterized by the interaction among bacterial, inflammation and genetic factors. Specific virulent oral microbials cause the host immune response in patients with a genetic predisposition. The inflammation of the periodontal tissues could result, if not properly treated, in clinical attachment loss (CAL), the formation of periodontal pockets and alveolar bone resorption, which could finally lead to tooth loss [2,3]. Recent studies showed that the immune system cells release cfDNA during periodontal inflammation to promote alveolar bone resorption [4,5]. For this reason, cfDNA has captured the attention of the periodontal area. 

Today, cell-free DNA (cfDNA) is commonly used as a biomarker in prenatal analysis and in the oncology field. Over the last years, cfDNA has caught the interest of scientists in other medical fields; for instance, cardiovascular disease [6], autoimmune diseases [7], sepsis [8], trauma [9] and others.

The term “liquid biopsy” specifies several body fluids that can be collected, such as blood and saliva. When high levels of biomarkers are found in saliva, they can be related to head and neck pathologies. Cell-free DNA (cfDNA) and mitochondrial cell-free DNA (mtDNA) are two markers that can be found in the body fluid of patients affected by oral conditions [10,11].

Several studies have found a greater concentration of cfDNA and mtDNA in the body fluids of patients affected by different cancer types than in healthy ones. For this reason, clinics have started to investigate the presence of the same circumstances in oral conditions, such as oral squamous cancer. Desai proposed the use of total cfDNA level as a screening marker for the early detection of oral precancer lesions and cancer [12]. A study conducted by Lin et al. [13] examined the cfDNA level in patients affected by oral squamous cell carcinoma (OSCC) compared to healthy ones. Their results showed that the cfDNA level was higher in patients affected by OSSC compared to the control group. These outcomes were similar to the results obtained in other studies that evaluated the same markers for solid cancers [14,15]. In particular, Lin et al. [13] obtained these outcomes in patients with extensive tumors, cervical lymph node metastasis and TNM (Tumor–Node–Metastasis) staging. The authors concluded that cfDNA is an independent indicator of cervical lymph node metastasis. According to these results, other researchers have investigated the concentration of these markers in the blood of patients affected by head and neck squamous cell carcinoma. Mazurek et al. [16] observed an increase of cfDNA in patients with N2-N3 lymph node metastasis affected by head and neck squamous cell carcinoma. They did not observe an increase in cfDNA level in patients with N0-N1 lymph node metastasis. 

Sayal et al. [17] evaluated the cfDNA and mtDNA levels in patients affected by head and neck squamous cell carcinomas (HNSCCs). They observed higher levels of cfDNA and mtDNA in patients affected by HNSCCs compared to healthy ones. Moreover, they evaluated how these two markers change their level in the case of oral leukoplakia (precancer oral lesion). From this evaluation, it also emerged that in precancerous oral lesions, the cfDNA and mtDNA level changed compared to the healthy patients. The authors claim that the variation in the concentration of these markers could be used to estimate the grade of epithelial dysplasia and for surveillance among patients [17]. A recent study conducted by Sayal et al. [18] shows that the level of mtDNA is correlated with survival in patients affected by HNSCC. The higher concentration of cfDNA and mtDNA in body fluid can also be associated with other conditions, including inflammation and infection [19]. Therefore, cfDNA and mtDNA concentrations can vary in periodontitis, considering it is an inflammatory disease. 

CfDNA was proposed as a new biomarker to study the disease’s evolution and progression in the periodontal field. A different concentration of cfDNA was seen among healthy patients, periodontally affected and gingivitis-affected patients [20]. Moreover, a recent study has shown therapeutic results in treating periodontitis in mice using nanoparticles that remove cfDNA [4]. The aim of this article is to report what was discovered about the relationship between cfDNA and periodontitis in the last few years. Additionally, it will underline the possible uses of cfDNA as a marker in periodontitis for diagnosis and therapy. 

## 2. Cell-Free DNA

In 1948, Mandel and Metais were the first to discover and describe cfDNA in the human plasma [21], as fragments of nucleic acids present in many fluids of the human body. The release of cfDNA is influenced by different variables such as age, smoking, physical exercise, sex, diet, infection, oxidative stress and pregnancy [22,23,24]. It originates from three main mechanisms:

- Apoptosis, in which the cell DNA is processed by endonucleases with the production of short fragments of DNA [25]. CfDNA originates from apoptosis and is formed by double-stranded fragments of about 150–200 base pairs [26].
- Necrosis is a mechanism of death common in cases of trauma and sepsis due to chemical or physical stimuli [27,28,29]. This mechanism [30] is correlated among Kilobase pairs of cfDNA [25]. cfDNA originating from necrosis has a longer length due to the increased time required to eliminate the necrotic cells, while the clearance of apoptotic cells requires less time [31].
- NETosis is a process that induces the neutrophil’s death after its contact with exogenous agents [25] and represents an active source of cfDNA. It is a particular process based on the release of traps (NETs, neutrophil extracellular traps) by neutrophils to contrast and kill microbes [32]. NETs are composed of histones and DNA. During the NET mechanisms, DNA is released in two forms: vital and suicidal NETosis. In their vital form, neutrophils release DNA and perform their phagocytic activity against pathogens [33,34], while in suicidal NETosis, the programmed death of neutrophils occurs after their contact with a pathogen [35,36].

CfDNA can be found in different forms; for instance, in free fragments, linked to proteins or packed in extracellular vesicles [37]. A cfDNA fragment is defined when there is DNA alone without other molecules [38].

It is possible to identify three types of cfDNA in the human fluid: endogenous nuclear DNA (or genomic DNA), mitochondrial DNA (mDNA) and bacteria (bDNA) or viral DNA [39,40]. Genomic DNA and mDNA can be transported by extracellular vesicles (EVs) [41]. Recently, EVs were discovered as another form of active cfDNA release [22]. These extracellular vesicles originated from the fusion of intraluminal microvesicles and can carry DNA both in their lumen and on their surfaces [42]. There are three main types of extracellular vesicles: exosomes, microvesicles and apoptotic bodies [43]. In EVs, it is possible to find genomic DNA, mitochondrial DNA, RNA, proteins and lipids [44]. Different studies showed that extracellular vesicles carrying DNA play different functions in cellular communication, immune system control, homeostasis and material transportation [45,46,47]. 

Normally, in a healthy patient, the concentration of cfDNA in the plasma is less than 10 ng for mL [48]. In cases of trauma, systemic disease, heart disease, cancer and inflammation, cfDNA concentration increases [49]. An endogenous source of cfDNA is the fetal cell-free DNA present in the blood of pregnant individuals. This type of cfDNA can be detected through a non-invasive procedure (non-invasive prenatal testing), and it can be used to identify a possible mutation in the fetus’s DNA. For example, using this marker, it is possible to search for the presence of trisomies [50]. In oncology cfDNA a blood sample can be used to search for a specific DNA methylation related to a specific cancer. Additionally, the analysis of specific DNA methylation present in the cancer’s cells shows the presence of recurrence after the conclusion of the treatment or which type of therapy is better [25,51].

To maintain homeostasis, the cfDNA produced is typically removed by the endonucleases, such as DNase I. Endonucleases are enzymes capable of deleting nucleic acids. A system is defined as healthy when a balance exists between the release of cfDNA and the removal of cfDNA. CfDNA is digested by DNases and removed from the bloodstream through the liver, kidneys and spleen. The correct and rapid removal of cfDNA avoids the occurrence of an inflammatory process [52]. DNases are present in blood and saliva [53,54]. When free DNA is linked with EVs or with other molecules, it is more resistant to the activity of DNases [38]. 

## 3. CfDNA and Periodontitis

The presence of hyperactive polymorph nucleate neutrophils (PNMs), which characterize periodontitis [55,56], induces the NETs system’s overstimulation. The NETs system, in the gingival sulcus of a healthy patient, allows the removal of bacterial, pathogen-associated molecular patterns (PAMPs) and damage-associated molecular patterns (DAMPs), releasing cfDNA and peptides [57,58]. DAMPs consist of intracellular components, such as proteins and nucleic acids, which the cells release during their necrosis process [59]. In the case of periodontitis, the level of the NETs system increases, causing the alteration of homeostasis and chronic inflammation [57,58]. 

Normally, during a proinflammatory immune process, the Toll-like receptors (TRLs) interact with different substances and allow the beginning of inflammation. In many inflammatory diseases, this process is altered, and TRL9 is involved [60]. It was discovered that the abnormal functioning of TRL9 plays a role in the development and establishment of periodontitis. An in vivo study showed that animals without TRL9 were resistant to the development of periodontitis. The same results were obtained in an in vitro study conducted by Kim et al. [61] and Crump et al. [62]. Moreover, a strong relationship was observed between TRL9 and periodontopathic bacteria [63]. TRL9 is one of the main receptors of cfDNA, and its interaction causes the beginning of the inflammatory process in alveolar bone inflammation [4]. TRL9 is normally present in the basement and sub-basement cells of the oral and pocket epitheliums [64]. In the periodontal pocket of patients affected by periodontitis, the TRL9 level is higher than that of healthy patients [63]. This discovery could be related to the active role of cfDNA in the development and progression of periodontitis [65]. In fact, a study conducted by Huang et al. [4] showed the relationship between high levels of cfDNA and elevated alveolar bone destruction in patients affected by periodontitis. In another study conducted by Huang et al. [66], it was found that cfDNA actively participates in bone resorption. The authors evaluated this activity by measuring the cfDNA level in GCF of patients during the post-operative 24 h in the sites where bone grafting was inserted. From the results of this evaluation, it was observed that the bone loss obtained after the alveolar bone grafting may be related to increased cfDNA levels in GCF [66]. 

**Figure 1:**
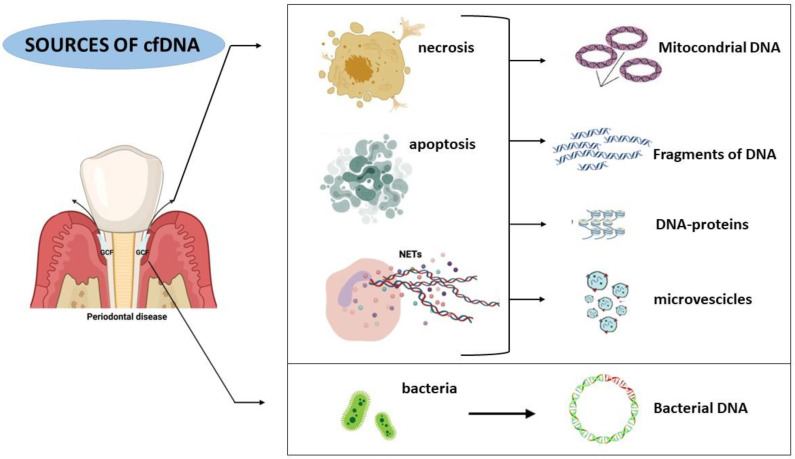
Description of the main mechanisms of origin of cfDNA in the organism and cfDNA’s structural characteristics that each mechanism produces.

The cfDNA in the mouth derivates from bacterial DNA (bDNA) [67,68], epithelial death cells of the periodontal tissue [69] and neutrophil extracellular traps (NETs) [70] (Figure 1). NETosis, in the mouth, physiologically avoids bacteria colonization on the gingival cells [71]. Excessive production of NETs interrupt homeostasis, allowing harmful periodontal bacterial entrance [57,58].

One of the sources of endogenous cfDNA is mtDNA. Correct mitochondrial function is fundamental for maintaining health. In the case of periodontitis, the dysfunction of mitochondria participates in the pathogenesis of the disease [72]. The role of dysfunctional mitochondria in the pathogenesis of periodontitis is probably related as mtDNA has similarities with bacterial DNA [73]. This characteristic underlines how mtDNA presence in the extracellular environment stimulates inflammation in many inflammation-related diseases [74]. In periodontitis, the presence of mtDNA in the extracellular space is caused by periodontal pathogens that stimulate the NETs activity [75,76]. In a study conducted by Liu et al. [74], it was demonstrated, for the first time, that in a culture of gingival fibroblasts affected by periodontitis, the exposition of periodontal pathogens has caused the release of mtDNA. Moreover, they observed an increase in mtDNA in mice affected by periodontitis compared to the control group [74]. In agreement with this study, it was proved that mtDNA outside the cells leads to bone resorption [77]. Furthermore, another study discovered that bDNA increases the extracellular mtDNA release, resulting in TRL9 activation [78]. 

As mentioned before, cfDNA is normally removed from liquids due to the presence of DNases. DNases are a group of enzymes that hydrolyze cfDNA, allowing its removal from the body fluids. It could be possible that the incorrect functioning of the salivary DNases is another reason why the cfDNA level increases in periodontitis [79]. It was seen that periodontal bacteria could influence the activities of DNases [80]. Only one study has evaluated the activity of salivary DNases in periodontitis patients. This study did not show differences in the activity of DNases between patients affected by periodontitis and healthy patients. These results could be linked to the fact that the samplings were frozen and stored [79]. Therefore, it is not yet clear whether there is a correlation with cfDNA concentration. For these reasons, further studies are necessary for a better understanding. Additionally, further evaluation should analyze the DNases activity immediately after the levy. 

**Figure 2:**
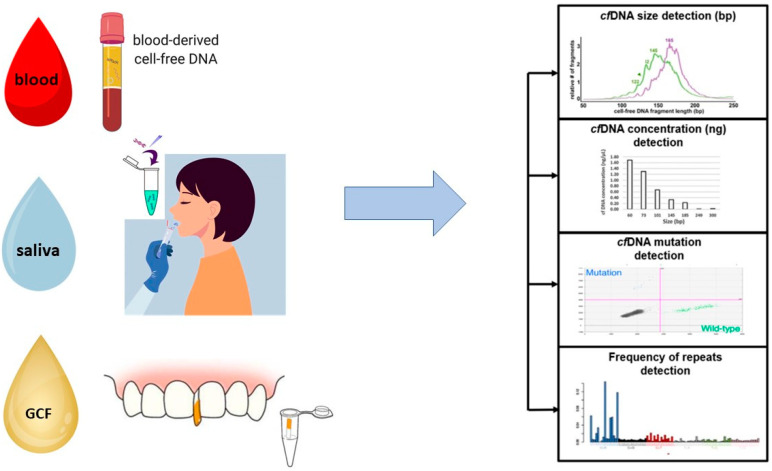
Description of the main methods of sampling biological fluids (blood, saliva and GCF) for research of the cfDNA level and of the analyses used for the study of cfDNA (cfDNA mutation, the variation of the cfDNA level and the size of the cfDNA). Partially modified and reproduced under permission of Creative Commons Licenses, from Hassan et al. [86].

CfDNA could be a new instrument to understand the status of periodontitis status. The short length of cfDNA can be a limit for its search, but due to different new technological methods, today, its detection is possible [25,81,82]. These methods are fluorescence [83], genomic sequencing [84] and polymerase chain reaction (PCR) [85]. In periodontology, cfDNA concentration was investigated in blood, saliva and gingival crevicular fluid (GCF) (Figure 2) [85]. Different studies have analyzed the level of cfDNA in periodontopathic patients and healthy patients in these different biofluids. 

### 3.1. Evaluation of the cfDNA Level in GCF

Two techniques can be used to collect gingival crevicular fluid (GCF): the washing technique [20,87] and the paper strips technique [88]. In the washing technique, the gingival pockets are washed with an isotonic solution, and the fluid that emerges from the pockets is aspirated. In the paper strips technique, three paper strips are used to collect the gingival crevicular fluid for 30 s each. Both techniques allow the collection of the GCF, but the paper strips technique is better because it does not require a long learning curve. In contrast, to collect GCF in the correct way with the washing technique, the operator needs to develop specific skills [89]. In a study conducted by Thaweboon et al. [89], GCF was collected for the evaluation of cfDNA in periodontopathic and healthy patients. It was seen that there was no statistical variation in the cfDNA concentration between the two techniques. Moreover, the authors observed little variation between these methods. The cfDNA level in patients affected by gingivitis was a little more concentrated using the paper strips than in the washing technique [89]. The same results were also obtained by Suwannagindra et al. [90]. Two studies evaluated the possible correlation between the cfDNA level in GCF and the periodontal clinical parameters (PD, BoP, PI plaque index). Suwannagindra et al. [90] collected the GCF in 20 patients affected by different degrees of periodontitis. After that, the concentration of cfDNA was evaluated, showing no correlation between the level of cfDNA and the periodontal parameters (PD, BoP and PI) [90]. In contrast, Zhu et al. [5] found a correlation between the level of cfDNA in GCF and periodontal parameters. The level of cfDNA increased based on the degree of the disease. In patients affected by gingivitis, the cfDNA levels were higher than in healthy patients, but in patients affected by periodontitis, the level of cfDNA was enhanced more than in patients affected by gingivitis. These results underline how the concentration of cfDNA in GCF is strongly correlated with the extent of the periodontal inflammation. Additionally, statistically predictive impacts of the cfDNA level in GCF and PD (pocket depth), BoP (bleeding on probing) and PI (plaque index) were shown [5]. The different results obtained from these two studies could be determined by the low number (only 20) of the patients evaluated in the study of Suwannagindra et al. [90] in contrast with the 114 patients in Zhu’s study [5] (Table 1). 

### 3.2. Evaluation of the cfDNA Level in Saliva 

In a sample of saliva, it is possible to detect a variety of biomarkers. Many factors, such as diet, disease and stress, can influence the elements of saliva [91]. In the periodontal field, using saliva allows us to evaluate the presence of cytokines, oxidative stress levels, antioxidants, periodontopathic bacteria and others [92,93,94,95,96,97]. The cfDNA in saliva comprises 70% of endogenous DNA and 30% of microbial DNA [98]. 

Zhun et al. [5] studied the cfDNA level variation in different biofluids, one of which was saliva. Their study showed that the cfDNA concentration was higher in patients affected by periodontitis and gingivitis compared to healthy patients. Moreover, they discovered a positive correlation between the cfDNA level and the clinical parameters; for instance, PD, BoP and PI [5]. Huang et al. [4] synthesized a type of nanoparticle to remove the cfDNA in the periodontal pockets to treat periodontitis in an animal model. To achieve their previous objective, they studied the mechanism between periodontitis and the increase of the cfDNA level in saliva and human blood. They observed that patients affected by periodontitis had a cfDNA concentration higher than patients affected by gingivitis or healthy ones. 

Konečná et al. [79] studied the salivary cfDNA concentration in 25 periodontopathic and 29 healthy patients. The results of this study showed that the total salivary cfDNA level in patients with periodontitis was higher than in healthy patients. Despite these initial results, when the saliva was centrifugated to remove cells, the level of cfDNA in both groups did not differ. This study has demonstrated that the level of mtDNA in saliva was higher in patients affected by periodontitis than in the healthy group [79]. This result underlines mitochondria’s important role in inflammation [99] (Table 1). Another study showed similar results in patients affected by periodontitis who had high cfDNA levels in saliva compared to the control group. Furthermore, in this study, the concentration of all the bacteria in saliva was not correlated with the salivary level of cfDNA in the periodontopathic patients [100].

It was recently discovered that methylation reaction products on the DNA chain can be detected in cfDNA. One of these methylation products is the global cytosine methylation (5 mC) found in breast, colorectal and prostate cancers and used as biomarkers [101]. Han et al. [102] researched the possible mutations of cfDNA that could be used in the periodontal field. They have researched the global epigenetic DNA present in the salivary small extracellular vesicle carrier DNA (sEVs) and in the genomic DNA (gDNA) among patients affected by gingivitis and periodontitis and healthy controls. The results showed that sEVs from the saliva of periodontopathic patients possess a significant increase of the 5 mC and m6dA (N6-methyl-2′-deoxyadenosine) methylation compared to the healthy group. The authors concluded that salivary sEV 5 mC methylation has a high sensitivity for discerning periodontitis patients from healthy ones [102]. However, further investigations are necessary on a huge cohort for a better understanding. The research of particular methylation in the cfDNA allowed us to understand from which tissue the cfDNA was released. Additionally, it was declared that the methylations present on the cfDNA are a valid method for cancer diagnosis [103,104].

### 3.3. Evaluation of cfDNA in Serum 

As mentioned, it is possible to detect cfDNA in blood. In fact, two preclinical studies showed increased cfDNA levels in plasma after the injection of *Porphyromonas gingivalis* (*P. gingivalis*) in mice [105,106]. A study by Zhu et al. [5] evaluated the level of cfDNA in the serum of patients with periodontitis and gingivitis and healthy patients. This study showed that the cfDNA concentration was higher in the blood of the periodontopathic patients, while the cfDNA blood level was not different between the patients affected by gingivitis and the healthy patients. The authors concluded by saying that these results showed how the degree of inflammation in the periodontal tissue influenced the cfDNA blood concentration [5]. Furthermore, another study obtained similar results by investigating the cfDNA level in serum between patients affected by periodontitis and healthy patients. At the end of the study, the periodontopathic patients had a higher level of cfDNA in serum compared to the healthy patients. Additionally, they were the first to prove the strong correlation between the cfDNA level in serum and GCF and the progression of periodontitis [4]. In a study by Liu et al. [74], mice affected by periodontitis had an enhanced mtDNA level in serum compared to healthy mice. The same mechanism was replicated in human gingival fibroblastic culture cells affected by periodontitis in which the release of mtDNA was seen. Moreover, human gingival fibroblast culture cells without periodontitis started to release mtDNA when exposed to *P. gingivalis* [74]. These results evidenced how mtDNA actively contributes to the development of periodontitis (Table 1).

Most of the studies evaluated biomarkers in saliva, blood and GCF on freezing samples. For this reason, it is important to underline that plasma freezing causes free DNA liberation from exosomes [107]. This is an aspect that must also be considered in salivary sampling because freezing is part of the processes used in many studies. 

### 3.4. Evaluation of cfDNA in Periodontitis and Oral Diseases

Many studies showed that cfDNA plays a central role in the development and progression of many diseases: for instance, rheumatoid arthritis, atherosclerosis and sepsis [65,108,109,110]. 

It can be seen that periodontal cfDNA is present in different biomaterials, such as serum [111], atherosclerotic plaque [112,113], synovial fluid [114] and intrauterine environment [115]. This underlines the possible relationship between periodontitis and systemic disease. 

Over the years, different studies have evaluated the link between periodontitis and arthritis. Both pathologies share common inflammation mechanisms and lead to bone loss [116]. In a study conducted by Oliveira et al. [117], the cfDNA levels in serum and saliva were evaluated among different groups of patients: patients affected by initial arthritis with and without periodontitis, patients affected by prior arthritis with and without periodontitis and healthy patients. The results of this study showed that patients affected by initial and prior arthritis associated with periodontitis possessed high levels of cfDNA in saliva and serum compared to the patients without periodontitis and arthritis. Both pathologies are characterized by the increase of NET activity, which feeds the chronic inflammation. When both pathologies coexist in the same patient, the NETs level is overstimulated, and the cfDNA level in saliva and serum is very high [117]. 

In recent decades, numerous studies have focused on the possible correlation between periodontitis and cardiovascular disease. Periodontitis is one of the main risk factors for cardiovascular disease [118,119]. According to these studies, bDNA of periodontal pathogens has been seen in serum and cardiovascular tissue [120]. A study conducted by Wu et al. [121] evaluated the presence of *P. gingivalis* DNA in the cfDNA of saliva and serum between patients affected by acute myocardial infarction (AMI) and patients without coronary heart disease. The authors of this study support the idea that periodontitis pathogens actively participate in developing atherosclerosis [119]. This study showed that patients affected by AMI were positive for *P. gingivalis* DNA in the cfDNA of the serum withdrawal. Moreover, the positivity with Pg is associated with the severity of coronary inflammation. Furthermore, there was no statistically significant variation between the positivity of *P. gingivalis* in the saliva cfDNA between the group of patients without cardiovascular disease and those affected by IMA. The authors concluded that the invasion and establishment of periodontal pathogens in the endothelial tissues were one of the risk factors for acute myocardial infarction. Additionally, the presence of Pg in the cardiovascular tissue increases the host response [121]. 

In pregnant patients with a predisposition to periodontitis, adverse outcomes were seen during the pregnancy. This is related to the presence of periodontal pathogens and the increase of immune stimulus caused by their presence. Different studies have shown the presence of periodontal pathogens DNA in the plasma of pregnant patients affected by periodontitis [115]. 

Diabetes is another disease in which NETs play a crucial role. NETs has an important role in the physiopathology of diabetes and periodontitis. Hyperglycemia causes the activation of the NETs mechanism. In a study conducted by Carestia et al. [122], an increase in the NETosis level was observed in patients affected by diabetes compared to healthy patients. For this reason, it would be interesting to analyze the cfDNA level in both pathologies and see how their coexistence impacts the cfDNA level.

## 4. Strategy to Treat Periodontitis Removing the cfDNA 

The discovery related to the presence of cfDNA in periodontitis has opened the possibility to a new therapy that uses cfDNA as a target, in particular for the treatment of bone loss [66] and inflammation in periodontitis. In a study conducted by Huang et al. [4], it was discovered that with the deletion of cfDNA in the periodontal pockets, the level of cfDNA in saliva and serum decreased. Furthermore, the intense relationship between the high level of cfDNA and elevated alveolar bone destruction was underlined. After this discovery, Huang et al. [4] formulated particular nanoparticles to remove cfDNA. These nanoparticles were firstly tested on cell cultures and subsequently on animals. The nanoparticles give better results when administered locally in the periodontal pockets instead of systemically. This study showed that the alveolar bone loss in the rats affected by periodontitis had a reduction. Further studies need to evaluate the efficacy and safety of these nanoparticles in humans. Despite the interesting results obtained, Huang’s study was the first and only one that created and tested a new drug that used cfDNA as a target in periodontitis. The presence of high levels of cfDNA in other inflammatory diseases has stimulated researchers’ interest in creating drugs against this target. Their results can lead to future studies about alternative treatments for periodontitis. 

Many studies showed that different cation nanoparticles can scavenge cfDNA. Liu et al. [123] developed nanoparticles to scavenge the increase of cfDNA in sepsis. The cfDNA-scavenging nanoparticles [20] were composed of cationic polyethyleneimine (PEI), which was loaded with zeolitic imidazolate framework-8 (PEI-g-ZIF). This nanosystem was tested in an in vitro model. The results of this study showed a decrease in the cfDNA, confirming the ability of these nanoparticles to bind and remove cfDNA in plasma [123]. In another study, a copolymer was developed and tested on animals as a new inhibitor of cfDNA in arthritis. The copolymer was composed of poly-lactic-co-glycolic acid (PLGA) and poly-2-diethylamino-ethyl methacrylate (PDMA). This copolymer in rats affected by arthritis was able to inhibit the TRL9 and scavenge cfDNA in plasma and inflamed joints. Although this copolymer’s positive results have been shown, it is necessary to carry out other investigations into its toxicity and efficacy [124]. Similar results were obtained by Pan et al. [125] during their study. In fact, it was seen that the inhibition of TRL9 allowed a decrease in periodontal inflammation in rats affected by arthritis [125]. Another study investigated Hexadimethrine bromide, a cation polymer that has an affinity with mtDNA. It was seen that this cation polymer allowed a decrease of the inflammatory mediators in a rat model [126]. Narayan et al. [63] observed that the high level of TRL9 in periodontal patients influenced periodontal tissue destruction.

The coexistence of cfDNA in the inflammation mechanism of different diseases can be an alternative target for treating patients affected by periodontitis with and without other diseases. 

## 5. Conclusions

CfDNA is a physiological biomaterial that has been observed to increase in several chronic inflammatory diseases, including periodontal disease. CfDNA actively participates in the pathology’s beginning and progression in periodontitis, favoring the inflammation’s continuation. The use of saliva and GCF to detect cfDNA are non-invasive methods that can be used for the future diagnosis and prognosis of periodontitis. The possibility of synthesizing nanoparticles which can remove cfDNA could be a new strategy to reduce the inflammation of periodontitis. Despite the promising results obtained about this new biomarker and new therapeutic targets in periodontitis, further studies are necessary to develop a reliable, safe and standardized protocol for the detection of cfDNA.

## Figures and Tables

**Table 1:** The table above summarizes the results obtained in the clinical studies which have analyzed cfDNA in different biofluids.

Source	Type of Study	Results	Ref.
GCF	Evaluation on humans	The periodontopathic patients showed a higher level of cfDNA compared to the control group	[89]
	Evaluation on humans	A higher level of cfDNA was observed in periodontopathic patients	[90]
	Evaluation on humans	It was seen that the level of cfDNA was correlated with the degree of inflammation in patients with gingivitis, periodontitis, and healthy patients	[5]
Salivary	Evaluation on humans	It was observed that the level of cfDNA reflected the degree of the inflammation based on the presence of gingivitis, periodontitis, or oral health	[5]
	Evaluation on humans	From this study, the results showed the presence of high levels of cfDNA in patients affected with periodontitis compared to the healthy patients	[79]
Serum	Evaluation on humans	The results showed that the blood level of cfDNA was higher in patients with periodontitis, while healthy patients and patients affected with gingivitis did not have variations	[5]
	Evaluation on mice	Mice affected by periodontitis presented high blood levels of mtDNA compared to the healthy mice	[74]

## Data Availability

Data are available from the corresponding author upon reasonable request.
